# Active Aging for Individuals with Parkinson's Disease: Definitions, Literature Review, and Models

**DOI:** 10.1155/2014/739718

**Published:** 2014-08-25

**Authors:** Seyed-Mohammad Fereshtehnejad, Johan Lökk

**Affiliations:** ^1^Division of Clinical Geriatrics, Department of Neurobiology, Care Sciences & Society (NVS), Karolinska Institutet, Novum 5th Floor, 14186 Stockholm, Sweden; ^2^Department of Geriatric Medicine, Karolinska University Hospital, 14186 Stockholm, Sweden

## Abstract

Active aging has been emerged to optimize different aspects of health opportunities during the aging process in order to enhance quality of life. Yet, most of the efforts are on normal aging and less attention has been paid for the elderly suffering from a chronic illness such as Parkinson's disease (PD). The aim of this review was to investigate how the concept of “active aging” fit for the elderly with PD and to propose a new model for them using the recent improvements in caring models and management approaches. For this purpose, biomedical databases have been assessed using relevant keywords to find out appropriate articles. Movement problems of PD affect physical activity, psychiatric symptoms lessen social communication, and cognitive impairment could worsen mental well-being in elderly with PD, all of which could lead to earlier retirement and poorer quality of life compared with healthy elderly. Based on the multisystematic nature of PD, a new “*Active Aging Model for Parkinson's Disease*” is proposed consisting of self-care, multidisciplinary and interdisciplinary care, palliative care, patient-centered care, and personalized care. These strategies could potentially help the individuals with PD to have a better management approach for their condition towards the concept of active aging.

## 1. Introduction

### 1.1. Background

Parkinson's disease (PD) is a progressive neurodegenerative disorder that affects 1% of all people over 60 years of age in industrialized countries [1] and is considered as the second most prevalent geriatric neurodegenerative disorder after Alzheimer's disease [2]. As a debilitating chronic condition, a high direct cost of 1.7 billion SEK has been estimated in patients with PD in Sweden [3]. PD is characterized by cardinal motor symptoms, including bradykinesia, rigidity, postural instability, and resting tremor. In addition to motor features, PD patients also suffer from nonmotor symptoms such as psychiatric problems, autonomic disturbances, pain, fatigue, and most importantly impaired cognition starting in executive functioning, memory, and spatial behavior during the early stage of the disease [4]. Later during the advanced stages, approximately 80% of PD patients developed dementia, which is an important predictor of mortality in PD population [5, 6]. The combination of different motor, nonmotor, and psychiatric features has made PD a unique chronic condition with lots of question marks and grey zones for patients' management. Motor disabilities could potentially restrict patients' physical activity and together with nonmotor symptoms worsen their quality of living during the lifespan [7]. Moreover, different problems in family life such as increased marital conflict, social isolation, loss of occupation, earlier retirement, and income loss may also affect their quality of life [8–10]. In parallel to the normal aging process, PD brings additional burden for both the patients and the caregivers, which lasts to the late elderhood. Although there is no worldwide consensus on the definition of elderly, the age of around 60 to 65 is considered to be the beginning of old age, which is roughly equivalent to retirement ages in most countries [11]. Since the average age at onset is 60, the majority of PD sufferers are among the elderly [12], which makes it necessary to adopt PD population with the new concepts on aging.

In the late 1990s, the World Health Organization (WHO) introduced the term “*active ageing*” as “the process of optimizing opportunities for health, participation, and security in order to enhance quality of life as people age” [13]. Since then, a large number of definitions have been launched by different authors [14], most of which have focused only on normal population and yet less attention has been paid to the elderly suffering from a chronic condition such as PD. While active ageing promotes elderly to realize their potentials for physical, social, and mental well-being throughout the life course and to participate in society [13], motor and nonmotor problems of PD necessitate a new adoption of “active aging” concept to fit the condition of the patients. In this view, the recently emerging caring approaches such as personalized medicine and patient-centered care have brought new insights into practice which will be further discussed in this review.

### 1.2. Aims

This review aims to investigate how the concept of “active aging” could be implementable for elderly with PD and to review the current literature and the knowledge gap in the field of active aging for the elderly population suffering from a neurodegenerative disease such as PD. Furthermore, we aimed to develop a new model of active aging for individuals with PD using the recent improvements in quality of care and management, namely, personalized care and patient-centered approaches.

## 2. Methods

As a narrative review article, relevant databases have been assessed to find out appropriate articles on active aging in neurodegenerative diseases and in particular Parkinson's disease. For this purpose, major electronic databases and citation indexes within the disciplines of medicine, healthcare, social science, and management including PUBMED/MEDLINE, EMBASE, and SCOPUS were searched with no date restriction. The main keywords consisted of “*active aging*” and “*Parkinson's disease*” which resulted in no hit. However, as there is no consensus on definition in the literature for active aging, the synonymous terms such as “*healthy aging,*” “*aging well,*” and “*successful aging*” were also used in the search strategy, which resulted in 47, 0, and 5 hits, respectively. After the screening process, none of these manuscripts were found relevant to the aims of this paper. Therefore, further search was performed purposefully focusing on different aspects of PD and active aging to find the literature that could provide relevant evidence for this topic. These aspects include “*physical activity,*” “*social communication,*” “*mental well-being,*” “*nutrition,*” “*quality of life,*” “*employment,*” “*lifelong learning,*” “*security,*” “*self-care,*” “*multidisciplinary care,*” “*palliative care,*” “*patient-centered care,*” and “*personalized care.*” Afterwards, the most relevant articles were selected. Reference and citation tracking of the selected papers for the final review were undertaken as the secondary searching process. Following reviewing of the literature, appraising of current evidences, and interpretation, key lessons were extracted that may lend direction for the implementation of the concepts of active aging in Parkinson's disease and development of a new model for this purpose.

## 3. Results and Discussion

**Figure 1:**
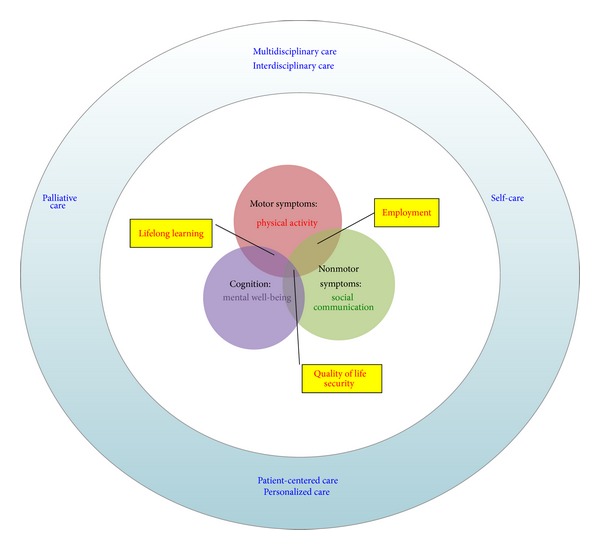
The schematic view of the platform to show how the symptoms of Parkinson's disease influence corresponding components of active aging, potential consequences (yellow boxes), and the proposed modifying caring approaches (surrounding blue circle) for adaptation.

After an inclusive review on the literature published until July 2014, we decided to create the concept of active aging in individuals with PD based on the most comprehensive and the highly accepted definitions per se. Since the unidimensional restriction of active ageing to a mere economic or physical framework has been shown to be problematic [14], we developed an “*Active Aging Model for Parkinson's Disease*” consisting of different aspects.  Figure 1 illustrates a summary of the framework including different symptoms of PD, corresponding aspect of active aging that is affected, and some possible modifying factors to adopt the model for individuals with PD. As it is shown, the main domains of active aging including physical activity, social communication, and mental well-being are affected by motor impairment, nonmotor symptoms, and cognitive decline, respectively. As a result, early retirement and/or unemployment could happen as a consequence of both motor and nonmotor disabilities, as for lifelong learning that is influenced by both motor and cognitive impairments. Finally, quality of life and security, as two main targets of the active aging concept, are affected by all problems of PD. In this framework, new caring approaches are depicted as supporting modifiers which could result in the adaptation of the “active aging” concept for individuals with PD.

Based on the WHO definition of active aging [13] and the literature review on recently proposed approaches for PD care, the “*Active Aging Model for Parkinson's Disease*” consists of the following:

- components:* physical activity, social communication, *and* mental well-being, *
- modifiers:* self-care, multidisciplinary care, palliative care, patient-centered care, *and* personalized care,*
- consequences:* quality of life (QoL), employment, lifelong learning, *and* security.*

This model is proposed with regard to the nature of PD, the symptoms and problems that the patients are dealing with, and the newest caring approaches fitted in the concept of active aging. As shown in Figure 1, symptoms of PD could directly affect all three main components of WHO's definition of active aging. As a progressive movement disorder, PD associates with restricted physical activity and the nonmotor symptoms such as depression, stigma, and anxiety could affect social communication of the patients. Later in the advanced stages of PD, cognitive impairment occurs which itself worsens mental well-being.

### 3.1. Components

#### 3.1.1. Physical Activity

The progressive motor impairment inclines many PD patients towards a sedentary life with low level of physical activity [15], which is not in line with the recommendations for active aging [13]. Obviously, increasing physical activity is an important health goal for individuals with PD in order to promote an active lifestyle [16]. However, gait disturbances, impaired balance, falls, and tremors make it difficult for PD patients to be physically active. The beneficial effects of several physiotherapeutic interventions on physical functioning, balance, and gait of the PD patients have been repeatedly shown [17, 18]; yet, these programs are not sufficient to achieve an active lifestyle especially during the advanced stages of PD when cognitive impairment also superimposes the activity barriers and leads to the sedentary condition [15, 19].

According to the concept of active aging, it is important to provide adequate protection, security, and care for PD patients when they need. Therefore, healthcare systems should have a plan creating a protective and secure evidence-based care for individuals with PD in order to help them improve their potentiality for physical activity. Recent evidences suggest that an individually tailored training program that has been adapted to patients' specific capacities and PD severity could potentially improve their physical activity towards active aging [20].

#### 3.1.2. Social Communication

Recent efforts have emphasized that active aging is not only the ability to be physically active but also continuing participation in spiritual and civic affairs in which social communication plays an important role. Individuals with PD might have difficulties in social communication through different perspectives. The PD-related spectrum of communication barriers contains somatic problems such as masking face, speech disturbances, and movement problems to spiritual issues as disease stigma and depression. Some PD patients have difficulties in their facial expressions that could lead to a profound impact on social interactions especially among the individuals with atypical forms of the disease such as progressive supranuclear palsy (PSP) [21]. On the other hand, the stigma attitudes and feelings of shame towards PD are other determinant factors to prohibit patients from socializing activities [22]. In addition to having an efficient management approach for the symptoms such as speech problems, masking face, and depression, it is of utmost importance to improve the attitudes of both patients and their surrounding persons towards PD. Educational meetings and programs for families, caregivers, and even the employers of the individuals with PD could help in avoiding stigmatizing behaviors and facilitating patients' involvement in social activities as an important part of active aging.

Recently, many patients with chronic conditions including the elderly with PD have shown a lot of motivations in communication with their peers and other individuals with similar condition. Through the aids of information technology, many patients are now socializing via the websites such as “Patients Like Me” (http://www.patientslikeme.com) sharing their experiences, emotions, and management plans with other patients. This could be a feasible and efficient initiative to motivate elderly individuals with PD having social communication through the process of active aging. Furthermore, volunteering activities could be another adapted way for social communication of these patients. Some patients are interested in events related to their chronic condition. One good example is the World Parkinson Congress (http://www.worldpdcongress.org/) where PD patients were actively involved as the members of the volunteers' team helping others in administrative affairs and informing them about the congress. They have the opportunity to communicate with other elderly with similar condition to improve their knowledge and insight, all of which could be in line with the concept of active aging.

#### 3.1.3. Mental Well-Being

Mental well-being is another component of active aging, which could be affected by neurodegenerative diseases such as PD. Point prevalence of dementia has been estimated as 40% in PD population [23], the condition which lately affects more than 75% of individuals with PD after 8 years [5] and more than 80% following 20 years of suffering from PD [6]. Even in nondemented PD elderly, a less severe condition, mild cognitive impairment, is common [24]. Early detection and proper management of cognitive impairment are crucial in individuals with PD in order to enhance their mental well-being in the concept of active aging. In addition to the evidence-based pharmaceutical treatments, other approaches such as behavioral therapy could be useful to manage cognitive problems in PD patients [25]. Lifetime educational programs, social activities, and using new approaches as multidisciplinary care could potentially postpone cognitive impairment and consequently improve mental well-being of PD patients. Spirituality can also give patients strength and improve their mental well-being. Recent research indicates that patients often rely on spirituality and religion to help in coping with physical illness and it is reason to believe that even healthy persons seek comfort in spirituality and religion. These are important to many individuals in the population and represent one of the most important influences in their lives. This may take different forms between different cultural and religious traditions [26]. Spirituality is used as a term to describe a sense of being often contributing to active aging. It could imply the purpose or the meaning in one's life or a guide of a higher power [27]. A review concluded that religious activity might have a positive impact on health outcomes [28]. This power might be in meditation, religion, or nature. Some find it in loving someone or feeling very loving towards others. It could be the challenge and reward of reaching beyond one's current limitations to seek and achieve something new. It might act as a help, catalyst, and mental support when making decisions and guiding the way in life. Regarding this, patients with a chronic condition such as PD should be aware of the fact that this probably will affect their attitudes, commitments, and beliefs as well as their assessments and judgments during aging process.

In another point of view, mental well-being could also strongly affect other aspects of elderly life in people with PD such as nutritional status. It has been shown that different commonly occurring mental and neuropsychiatric symptoms in PD such as depression, anxiety, dementia, confusion, and apathy contribute to decreased food intake and subsequent weight loss in elderly patients with PD [29]. Not only mental well-being, but also other components of “active aging” concept, namely, physical activity and social communication, influence nutritional status in PD patients, since motor, nonmotor, and cognitive symptoms all go hand in hand with nutrition. Evidence has shown that malnutrition is prevalent in PD patients and risk of malnutrition is even higher [29].

### 3.2. Consequences

#### 3.2.1. Employment

Evidences show higher rate of unemployment and earlier retirement in individuals with PD compared to healthy elderly in various societies [30, 31]. In one study the median age of retirement in individuals with PD was shown to be 58 yr and 61 yr for males and females, respectively, which was 4 to 5.5 yr lower than the corresponding ages in other elderly [9]. Another study showed that less than 16% of the PD patients had worked for more than five years after diagnosis [32]. Sometimes, this loss of ability to work is in parallel with the loss of independency in living among the patients due to the progression of disease severity in the older age [33]. Nevertheless, in some other occasions, this early retirement could be a sign of the so-called “*disablism*” or disability discrimination in addition to the “*agism*” itself. In a vicious cycle, loss of employment could potentially induce social and economic burden for the individuals with PD, which distract them from their social networks, occupational skills, and spiritual involvements. It has been shown that severity of PD symptoms, lack of support in the workplace, and opportunities for early retirement were the factors affecting withdrawal from the labor market among the PD population. On the other hand, age at diagnosis, support received from employers, and manipulation of drug therapy were associated with maintaining their employment [32].

Patients, their families, and their employers must adjust the working environment and use a coping strategy in order to adapt to their new condition. There is an extreme need for positive action to support people with progressive conditions (i.e., PD) who intend to continue their labor in the workplace, which is in line with the concepts of active aging.

#### 3.2.2. Quality of Life

One ultimate purpose of the call for active aging is to improve the quality of life for the elderly. As a result, it seems necessary to pay attention to the factors affecting QoL in elderly suffering from chronic diseases like PD. Higher severity of motor disability; longer duration of disease [34]; nonmotor symptoms, such as depression, sleep disorders, and fatigue [35]; mood, gastrointestinal, and urinary disorders [36]; and nutritional status [37] were all demonstrated to associate with poorer QoL in individuals with PD. Knowing these determinant factors, comprehensive interventions are needed to improve the QoL in elderly with PD. Evidences have shown that multidisciplinary interventions could be beneficial to enhance QoL in PD patients such as adding cognitive-behavioral strategies and physiotherapeutic plans [38] to their routine medications.

### 3.3. Modifiers

There are some recently promoted strategies that could potentially help the elderly with a chronic disease such as PD to have a better management approach for their condition towards the concept of active aging.

#### 3.3.1. Self-Care

Considering the visions of the active aging concept, it is clear that visiting patients with chronic diseases only every several months could not be helpful, especially when it comes to PD with a highly stochastic clinical presentation. As a result, there is an extreme need to develop a new model of caring approach for PD that copes with the concepts of active aging. Recently, the middle-range theory of self-care for chronic illnesses has been introduced, which contains three main domains: self-care maintenance, self-care monitoring, and self-care management [39]. In the context of PD, self-care maintenance reflects in patients' behaviors to maintain physical and emotional stability, which is promotable through patients' education and enhances their insight into the motor and nonmotor parts of PD. Self-care monitoring refers to the process of observing and preferably recording oneself for the fluctuations in signs and symptoms of PD. Nowadays, there is an increasing intention among the PD community towards self-measurement of the symptoms and tracking of the changes in the severity of motor problems such as tremor. A growing number of gadgets and tools are being developed to help PD patients in self-monitoring of their symptoms such as mobile apps, computer-based software, charts, and diaries [40]. After self-measurement, gathered information is used in self-care management with appropriate timely responses to the fluctuations in signs and symptoms. The ultimate vision of this self-care approach is to dynamically manage PD that has certain benefits for the elderly suffering from this disease. Nonetheless, it must be noted that such a pragmatic approach still needs to be supported by research-based evidences for the healthcare decision makers on one side and increased health literacy in the patients' side as well. Self-care can optimize opportunities for health in elderly with chronic illnesses, which is in line with the concept of active aging.

#### 3.3.2. Multidisciplinary and Interdisciplinary Care

As the definition of active aging itself addresses multiple disciplines of human health, multidisciplinary approach of care is an undeniable part of modern management for the elderly with chronic diseases. Multidimensional approaches help to raise alertness regarding the various domains of PD consisting of motor and nonmotor symptoms through which patients can age actively. PD is a very tangible example of a multidimensional chronic disease, where both somatic and psychotic aspects of the elderly health are involved. The combination of motor and nonmotor symptoms together with some general aspects of daily life (i.e., fatigue, pain, and malnutrition) necessitates a multidisciplinary approach to manage the care for individuals with PD.

Interdisciplinary collaboration has been proposed as an important strategy to boost coordination of healthcare system [41], by integration of multiple disciplines to move towards interdisciplinary approach to patients' care especially in case of chronic diseases such as PD. Elderly people with PD could benefit from interdisciplinary caring approach since numerous evidences have shown the advantages of multidisciplinary interventions such as cognitive behavioral therapy [42].

#### 3.3.3. Palliative Care

In line with the multidisciplinary and interdisciplinary approaches to provide better care services for PD patients, palliative care has emerged. Palliative care for elderly with PD should include a multidisciplinary team of physicians, nurses, physiotherapists, occupational therapist, speech therapists, dieticians, psychologists, social workers, caregivers, and even family members with an interdisciplinary approach to treat, care, and help patients maintain their dignity throughout the disease course [43]. According to the new management models for PD, palliative care is not restricted to the end-of-life care [44–46] and patients could benefit earlier from palliative care during the mild stages as well [43]. Early installation of palliative care improves QoL in elderly with PD which makes it a needed strategy towards the adaptation of the concept of active aging for individuals with PD.

#### 3.3.4. Patient-Centered Care

The modern definition of active aging has a prominent focus on the role of individuals to whom the concept is applying. Each elderly individual is encouraged to realize his/her potential for physical, social, and mental well-being throughout the life course [13]. Consequently, elderly patients should also have a central determining role in their caring approaches in order to have an active aging in parallel with their chronic condition. In other words, patient-centered care is a key concept to implement active aging for individuals with chronic illnesses including PD. Nonetheless, a transformative shift in the understanding of healthcare providers is required to believe what patients are capable of in order to implement the patient-centered approach and improve the quality of healthcare for PD patients [47]. For an efficient model, PD patients, their family, and caregivers should receive necessary education and information for being actively involved in their caring approach and not just passively put aside. Giving PD patients and their caregivers a central position as health coaches can play a considerable role in meeting their needs [47], which could ultimately improve their QoL through an active aging process.

#### 3.3.5. Personalized Care

As a chain of concepts, self-care, interdisciplinary, and patient-centered approaches are accordingly completed with the new idea of personalized care. PD is a debilitating lifelong condition with lots of fluctuations and variations in the trend, response to medication, prominent symptoms, and course of the disease. As said by Wicks et al. no two patients are alike in different aspects of the disease which makes PD an inappropriate entity to have a “*one size fits all*” caring approach [40]. Nowadays, an innovative approach is growing to adjust the treatment algorithm and caring approach based on the specific characteristics and needs of each patient, which is called “personalized approach.” In line with the concept of active aging, personalized care could help elderly with PD to cover more of their needs, receive more efficient treatments for different aspects of their disease, and implement a specific interdisciplinary caring protocol to improve their QoL and cope with their capacities.

## 4. Conclusion

With regard to the nature of PD, several adjustments are required to adapt the caring approaches of the elderly with PD in order to implement the concept of active aging for them. In this paper, a new framework is proposed, helping the achievement of active aging model for individuals with PD. Our literature review showed that main domains of active aging including physical activity, social communication, and mental well-being are affected in PD. Based on recent improvements in caring approaches for persons suffering from chronic illnesses, self-care, multidisciplinary and interdisciplinary care, palliative care, patient-centered care, and personalized care could be helpful as the means for the implementation of active aging for the elderly with PD. Presented as modifying elements in this paper, these modern caring approaches are interconnected to each other and act as a chain of relationships. Interdisciplinary approach motivates a collaborative integration between various healthcare professionals to provide palliative care in a multidisciplinary team working to cover not only the motoric symptoms of PD but also psychiatric and other nonmotor symptoms. In interdisciplinary approach more attention is paid to the central role of the patients' participation in their care where the assessments and management are guided through a self-care algorithm, which eventually opens the door for the new concept of personalized care. This paper highlights the need for special action from the patients themselves, their caregivers, and their family members, as well as the healthcare professionals, for more efficient care approaches to adapt the concept of active aging in individuals with a chronic illness such as PD.
